# A systematic review and quality appraisal of guidelines and recommendations for home enteral tube feeding in adults

**DOI:** 10.1038/s41430-024-01500-1

**Published:** 2024-09-03

**Authors:** Andriana Korai, Isabella Thomson, Sharon Carey, Margaret Allman-Farinelli

**Affiliations:** 1 Nutrition and Dietetics Group, Sydney Nursing School, Faculty of Medicine and Health, The University of Sydney, Camperdown, NSW Australia; 2 Charles Perkins Centre, The University of Sydney, Camperdown, NSW Australia; 3 Department of Nutrition and Dietetics, Royal Prince Alfred Hospital, Camperdown, NSW Australia

**Keywords:** Nutrition, Health policy

## Abstract

Home Enteral Tube Feeding (HETF) is a viable option for people within primary care settings when oral intake is insufficient to meet nutritional needs. As HETF is not a risk-free therapy, guidelines exist to enable its safe provision. This review aims to summarise existing guidelines and their recommendations pertaining to the provision of HETF and appraise their methodological quality. A systematic review was conducted according to the Cochrane Handbook for Systematic Reviews, PRISMA-checklist and a 2019 methodological guide specific to the review of clinical practice guidelines (PROSPERO registration: CRD42023456223). Records were sourced from five bibliographical databases (Medline, Embase, PsychINFO, Scopus, Cinahl) and the grey literature (64 websites, seven guideline repositories). The AGREE-II tool was applied to eligible guidelines. The recommendations of guidelines meeting a predetermined threshold score (domain 3 ‘rigour of development’ score >70%) were extracted, grouped, and assessed using the AGREE-REX tool. A total of 2707 records were screened with 15 guidelines meeting eligibility criteria. The median (IQR) overall AGREE-II score (/7) of all guidelines was 3 (3–5) and only 3/15 guidelines achieved a domain 3 score >70%. The median (IQR) overall AGREE-REX score was 33% (26–37%). No recommendation group achieved a domain score above 70%. No guideline or recommendation group was suggested for use without modification. Key limitations included suboptimal stakeholder involvement and implementability, and lack of methodological transparency. Current HETF guidelines inadequately align with methodological standards. This review highlights key areas HETF guideline developers should consider to create more relevant and implementable guidelines.

## Introduction

Enteral nutrition (EN) enables optimisation or preservation of nutritional status in individuals with malnutrition or risk thereof and with compromised oral intake. Indications for the initiation of EN may include increased nutritional requirements, gastrointestinal tract malignancies, reduced nutrient absorption due to inflammatory conditions or swallowing difficulties arising from neurological disorders [1]. EN is mostly administered through nasoenteric or the stomal route, where percutaneous feeding devices can be inserted endoscopically, radiographically, or surgically [2, 3]. While EN can be provided across the continuum of care, home enteral tube feeding (HETF) specifically involves delivering liquid nutrition via one of these routes within a domiciliary care setting [4]. While the cost-effectiveness of HETF compared to treatment in hospital has not been thoroughly evaluated, cost savings analyses have shown savings in HETF sub-groups, and across the care continuum in England of up to £65,484,550 for all forms of nutrition support inclusive of HETF [5, 6].

Attempts have been made to estimate the global point prevalence of people receiving HETF through large-scale surveys and retrospective studies as national registries are limited [7]. In the last five years, national prevalence has only been reported in Australia and New Zealand (234 HETF patients per million) and Poland has reported total HETF cases, although both used clinician-administered surveys which are prone to underestimation [7, 8]. Challenges with provision of HETF have been reported internationally in terms of clinical complications, funding and organisation of services, supply of consumables and research and development [9, 10]. This is reflected in the significant variability noted in the provision of care to HETF patients on a national and global level [7, 11]. The need for comprehensive clinical guidelines which additionally address barriers and facilitators to providing optimal care to this population group have been advocated [11]. Guidelines from well-known professional societies and government agencies have widespread use by clinicians caring for people receiving HETF [12, 13], however, all available guidelines have not previously been collated or assessed for their quality.

Multiple standards and guidance for development of high-quality clinical practice guidelines exist [14]. The development of evidence-based recommendations alone is insufficient to produce a high-quality guideline as guidelines should be outcome focused, capable of adaptation to various global audiences, frequently updated and involve collaboration between all relevant stakeholders including consumer-led expert opinion [14]. Groups such as the Scottish Intercollegiate Guidelines Network and the Guidelines International Network have endorsed use of the tools produced by the Appraisal of Guidelines REsearch and Evaluation (AGREE) collaboration to evaluate the quality of clinical practice guidelines [15, 16]. This review aimed to systematically identify and summarise existing guidelines pertaining to the provision of HETF in adults and assess the quality of guidelines and their recommendations using internationally recognised quality assessment tools.

## Methods

A protocol for this systematic review of guidelines was developed a priori and registered with PROSPERO (identification CRD42023456223) in accordance with the Preferred Reporting Items for Systematic Reviews and Meta-Analyses (PRISMA) statement [17]. A protocol amendment was submitted (12^th^ December 2024) with changes to the grey literature sources searched and eligibility criteria after piloting the study selection process. Further detail for the data extraction plan was also provided. This review was also informed by the Cochrane Handbook for Systematic Reviews of Interventions and Johnston et al. methodological guide for systematic reviews of clinical practice guidelines [18, 19].

### Literature search

Five bibliographical databases (MEDLINE, EMBASE and PsycInfo (OVID interface), CINAHL, and Scopus) were searched for published eligible guidelines on HETF on September 26, 2023. A search strategy was developed for Medline and translated across the remaining four databases (Table S1).

Additionally a grey literature search including seven guideline repositories and 64 key government health agency and nutrition association websites was conducted on September 29, 2023. The choice of guideline repository was based on Cochrane recommendations and the websites included were informed by a preliminary Google search and content experts on the research team. A summary of the sources and search methods used is provided in Table S2. The reference lists of included guidelines were also hand-searched to identify additional potentially eligible guidelines.

### Eligibility criteria

Included guidelines contained a dedicated set of recommendations specific to the provision of HETF to people aged over 18 years old. Guidelines covering any aspect or stage of the patient journey associated with HETF, from initiation to management and discontinuation were included. These had to be explicitly identified as a guide, guideline, standard or recommendations. Guidelines had to be evidence-based and/or developed through consensus, although could be presented in any format and with any intended end-user. Records providing inadequate methodological detail to ascertain whether evidence-based and/or consensus methods were used were excluded. Guidelines pertaining exclusively to short term enteral feeding or where the duration of feeding could not be determined were excluded, as where guidelines for specific diseases or conditions unless they specifically addressed HETF. Narrative reviews and opinion pieces were excluded. Guidelines were included if they were available in English and were produced from 2000 onwards to ensure currency.

### Study selection

All bibliographical records were imported into the reference management software EndNote (Version 20, Clarivate Philadelphia) where duplicates were removed as previously described [20]. The remaining records were imported into Covidence Systematic Review Software (Veritas Health Innovation, Australia 2023) for two-step screening conducted independently by two reviewers (AK, IT). Titles and abstracts were screened against eligibility criteria. Full-texts of records deemed eligible from this step were then obtained and screened. When consensus could not be reached between the two reviewers, conflicts were resolved through discussion with a third reviewer (SC). Where multiple editions existed, the most recent guideline was selected.

### Data extraction and quality assessment of guidelines

A data extraction form was created in Excel (Microsoft Corporation Version 16.77.1, 2023) to collate pertinent information from eligible guidelines. Information extracted included: guideline title, authorship, publication and update year, applicable geographical region, any affiliated agencies or associations, the aspect(s) of HETF considered, the applicable population group, whether a systematic search was conducted, methods used to evaluate the body of evidence and what consensus method was used.

Quality appraisal was completed with the AGREE-II tool. This tool evaluates the methodological rigour of guideline development through 23 items organised into six domains, where each item is rated against specific criteria and considerations using a 7-point grading system (1 – strongly disagree to 7 – strongly agree). Guidelines are then assigned an overall assessment (1 – lowest possible quality to 7 – highest possible quality) accompanied by a statement on recommended use which considers the overall quality of the guideline and its appropriateness for use in practice [21].

Supporting documents including previous and abridged versions, quick reference guides, technical reports, methodological manuals, guideline development policy statements, standard operating procedures and supplementary materials were retrieved prior to quality appraisal. Where applicable, corresponding authors were contacted twice to request access to supporting documents cited in the guideline but not publicly available. If no response was received quality appraisal was conducted without the additional documents.

Data extraction and quality appraisal using AGREE-II was conducted concurrently by two independent reviewers (AK, IT). All appraisers were trained to apply the AGREE-II tool by studying the manual and relevant publications [21, 22]. Application of the tool was also discussed prior to its use by the research team to promote consistency in how the tool was applied (MAF, AK, IT). For AGREE-II items which were not applicable to a guideline a rating of 1 (absence of information) was provided and this score was contexualised as recommended in the AGREE-II manual [21]. ‘Recommendations for use’ were informed by both the quality assessment and the availability of alternative guidelines. Differences in scores were resolved through discussion and final item scores and overall assessments reached through consensus. An experienced user of the tool (SC) then reviewed consensus scores from items where the two appraisers’ scores differed by >1. Consensus is an appropriate approach when less than four appraisers are available and has been previously employed [23, 24]

Scaled domain scores were calculated using consensus scores and the formula: (obtained consensus score – minimum possible score)/(maximum possible score – minimum possible score) [21]. As the AGREE-II user manual does not define quality cut-off scores, high quality guidelines were considered those with domain 3 ‘rigour of development’ scaled scores ≥70% as a high score in this domain is indicative of high methodological quality and transparency in reporting [25]. The quality of recommendations from high quality guidelines were then assessed using the AGREE-Recommendation EXcellence (AGREE-REX) tool [26].

### Data extraction and quality assessment of recommendations

The AGREE-REX tool assesses the quality of recommendations made by guidelines as determined by their clinical credibility, trustworthiness and implementability. The tool consists of nine items categorised into three domains. Each item consists of two evaluations, the first is informed by whether the recommendation(s) considered the criteria relevant to each item in their development, and the second optional evaluation rates the suitability of the recommendation(s) to a particular setting. Both evaluations use a 7-point grading system (1 – strongly disagree to 7 – strongly agree). For this quality appraisal only the first evaluation was completed as the results were not intended to guide adoption in a particular setting.

A second data extraction form was created in Excel (Microsoft Corporation Version 16.77.1, 2023) to collate all recommendations related to care of people receiving HETF from the included guidelines and their assigned grading. Relevant recommendations were extracted by one researcher (AK) and reviewed by a second (SC). The AGREE-REX tool was then applied to clusters of recommendations addressing a similar HETF topic within each included guideline. This approach was taken as it was believed that quality may vary between recommendations given the widely reported scarcity of high-quality research in this area and resource constraints made it unfeasible to assess each recommendation separately [26]. Six clusters were used, adapted from the categorisations used in the ESPEN Home Enteral Nutrition guideline [4], which was the sole high-quality guideline dedicated explicitly to HETF, and the nutrition care process [27]. Clusters included: commencement of HETF; care of feeding site and enteral access devices; recommendations for feeding; monitoring HETF; termination of HETF and requirements of HETF services. Additional detail for the clusters used is provided in Table S3. AGREE-REX assessment was independently completed by two reviewers (AK, IT) and final scores were agreed through consensus. Both assessors studied the AGREE-REX manual, and the tool was piloted on one cluster for a single guideline and results discussed prior to completing the remaining assessments. Scaled domain and overall scores were calculated using consensus scores and the formula: (obtained consensus score – minimum possible score)/(maximum possible score – minimum possible score) [26]. All data extraction forms and extracted data will be made available upon reasonable request.

### Data synthesis

Guideline characteristics were summarised descriptively. Domain scores were presented as percentages (scaled scores) and overall assessments as a score of seven for AGREE-II and as a scaled percentage for AGREE-REX. Overall assessments were accompanied by a statement on the assessors’ recommendations for use. Item, domain and overall scores were summarised using descriptive statistics (median with interquartile range (IQR)) for both tools across all eligible guidelines. Median and range was used only when comparing AGREE-REX scaled domain scores across recommendation clusters as only two to three scores were being summarised. Domain results from both tools were also presented as colour-coded quartiles and recommendation gradings were presented as proportions. All analyses were carried out using Excel (Microsoft Corporation Version 16.77.1, 2023).

## Results

### Study selection

**Fig. 1:**
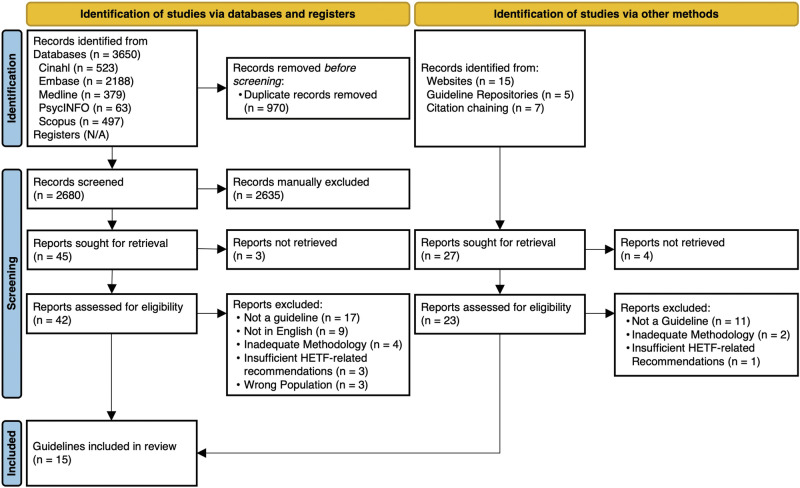
PRISMA flow diagram outlining study selection process [17]. A total of 2707 unique records were identified from all sources, of which 15 were included.

The database search identified 3650 records. A total of 970 duplicates were removed and 2680 records were screened based on their title and abstract. Following exclusion of 2635 records, 42 records were successfully retrieved for full-text screening and six guidelines were included. The grey literature search identified an additional 20 records from websites and guideline repositories, of which seven met the eligibility criteria. Seven additional records identified from citation chaining of included guidelines were screened of which two met eligibility criteria. A total of 15 guidelines were included in this review. The PRISMA flow diagram outlining the selection process is shown in Fig. 1.

### Guideline characteristics

All guidelines were produced by professional associations or government agencies. The guidelines originated from the USA (*n* = 4), Europe (*n* = 3), the UK (*n* = 3), Australia (*n* = 2), France (*n* = 1), Italy (*n* = 1) and Korea (*n* = 1). The primary focus of most guidelines (*n* = 10) was clinical management of one or more methods of nutrition support. Of these, three were exclusive to HETF [4, 28, 29], two covered enteral nutrition more broadly [30, 31], one addressed enteral and parenteral nutrition [32] and four encompassed all forms of nutrition support [12, 33–35]. Four guidelines were dedicated to care of feeding sites and enteral access devices [36–40] and two studies focused on organisation of services providing care to people receiving HETF [29, 41]. Seven of the guidelines included children in the target population, while exclusion of children was unclear in two guidelines. Eight of the guidelines employed a systematic literature search as part of their methodology and nine used standardised evidence grading systems. Four guidelines consistently used and clearly described a structured approach to reach consensus of recommendations. All guideline characteristics are outlined in Table 1.

**Table 1:** Characteristics of home enteral tube feeding guidelines.

Title	Authors	Year Published (Last Updated)	Location	Association/ Agency	Focus of Guideline	Population	Systematic Search	Evidence Evaluation Method	Consensus method
Clinical Practice Guidelines for Percutaneous Endoscopic Gastrostomy [36]	Tae et al.	2023	Korea	KSGE	Care of Feeding Site and Devices	Adults (inclusion of children unclear)	Yes	GRADE	Structured Process
Endoscopic Management of Enteral Tubes in Adult Patients (Part 1 and 2) [37, 38]	Arvanitakis et al.	2021	Europe	ESGE	Care of Feeding Site and Devices	Adults	Yes	GRADE	Unclear
ESPEN Guideline on Home Enteral Nutrition [4]	Bischoff et al.	2020	Europe	ESPEN	Clinical Management of HETF	Adults (inclusion of children unclear)	Yes	SIGN	Structured process
ESPEN Guideline on Clinical Nutrition and Hydration in Geriatrics [35]	Volkert et al.	2019	Europe	ESPEN	Provision of Nutrition Support Therapies	Elderly ( > 65 years old)	Yes	SIGN	Structured Process
Clinical Practice Guidelines for the Nursing Management of Percutaneous Endoscopic Gastrostomy and Jejunostomy (PEG/PEJ) in Adult Patients [39]	Roveron et al.	2018	Italy	AIOSS, ANIGEA, ANOTE	Care of Feeding Site and Devices	Adults	Yes	SIGN	Informal / Unclear
Nutrition Support for Adults: Oral Nutrition Support, Enteral Tube Feeding and Parenteral Nutrition [12]	N/A	2006 (2017)	UK	NICE	Provision of Nutrition Support Therapies	Adults	Yes	SIGN	Informal and Structured processes
ASPEN Safe Practices for Enteral Nutrition Therapy [31]	Boullata et al.	2017	USA	ASPEN	Clinical Management of Enteral Nutrition	Adults and Children	Unclear	Informal	Informal
Enteral Tube Feeding for Individuals with Cystic Fibrosis: Cystic Fibrosis Foundation Evidence-Informed Guidelines [30]	Schwarzenberg et al.	2016	USA	ECFS	Clinical Management of Enteral Nutrition	Adults and Children with Cystic Fibrosis	Yes	Nil	Structured Process
A Clinician’s Guide: Caring for People with Gastrostomy Tubes and Devices [40]	N/A	2014 (2015)	Australia	NSW ACI, GENCA	Care of Feeding Site and Devices	Adults and Children	Yes	NHMRC	Informal
ASPEN Standards for Nutrition Support Home and Alternate Site Care [34]	Durfee et al.	2001 (2014)	USA	ASPEN	Provision of Nutrition Support Therapies	Adults and Children	No	Nil	Informal/ Unclear
Guidelines for Home Enteral Nutrition (HEN) Services [29]	Ward et al.	2007 (2012)	Australia	NSW ACI	Clinical Management of HETF, Organisation of HETF Services	Adults and Children	Unclear	Nil	Informal
Clinical Practice Guidelines from the French Health High Authority: Nutritional Support Strategy in Protein-Energy Malnutrition in the Elderly [33]	Raynaud-Simon et al.	2011	France	HAS	Provision of Nutrition Support Therapies	Elderly ( > 70 years old)	Unclear	HAS method^a^	Informal / Unclear
The Provision of a Percutaneously Placed Enteral Tube Feeding Service [41]	Westaby et al.	2010	UK	BSG	Organisation of PEG Services	Adults and Children	No	Canadian Task Force method (adapted)^b^	Unclear
Guidelines for the Management of Enteral Tube Feeding in Adults [28]	Holmes et a.	2004	UK (Northern Ireland)	CREST	Clinical Management of HETF	Adults	No	Nil	Informal / Unclear
Guidelines for the use of Parenteral and Enteral Nutrition in Adult and Pediatric Patients [32]	August et al.	2002	USA	ASPEN	Clinical Management of Enteral and Parenteral Nutrition	Adults and Children	No	Unclear^c^	Informal

*AIOSS* Italian Association of Stoma care Nurses, *ANIGEA* Italian Association of Gastroenterology Nurses and Associates, *ANOTE* Italian Association of Endoscopic Operators, *ASPEN* American Society for Parenteral and Enteral Nutrition, *BSG* British Society of Gastroenterology, *CREST* Clinical Resource Efficiency Support Team, *ECFS* European Cystic Fibrosis Society, *ESGE* European Society of Gastrointestinal Endoscopy, *ESPEN* European Society for Clinical Nutrition and Metabolism, *GENCA* Gastroenterological Nurses College of Australia, *GRADE* Grading of Recommendations Assessment, Development and Evaluation, *HAS* Haute Autorité de santé (French National Authority for Health), *KSGE* Korean Society of Gastrointestinal Endoscopy, *NHMRC* National Health and Medical Research Council, *NICE* National Institute for Health and Care Excellence, *NSW ACI* New South Wales Agency for Clinical Innovation, *SIGN* Scottish Intercollegiate Guidelines Network, *UK* United Kingdom, *USA* United States of America.

^a^A four-level system used to categorise the strength of each included evidence source, this then informed grading of recommendations. Evidence type with some consideration of risk of bias.

^b^A six-level system used to categorise the strength of each included evidence source. Focus on evidence type with minimal consideration of risk of bias.

^c^Recommendations were graded using an adapted version of that previously employed by the Agency for Healthcare Research and Quality which considered study design, however, it is unclear if a structured approach was taken to grading the quality of each evidence source included.

### Assessment of guidelines using the AGREE-II tool

AGREE-II quality scores of the 15 guidelines included are presented in Table 2. The median overall quality score out of 7 for all guidelines was 3 [3–5]. The NICE guideline on nutrition support for adults was the highest scoring guideline (6/7) both in terms of overall quality and across all six domains. The highest scoring domain across all guidelines was ‘clarity of presentation’ (83% [64–86%]) and the lowest scoring domain was ‘applicability’ (8% [2–15%]). Of all domain scores, 23% (21/90) had scaled domain scores ≥75% and 29% (26/90) had scaled domain scores <25%. The highest rated item across all guidelines was item 16 ‘the different options for management of the condition or health issue are clearly presented’ (domain 4) (7 [6–7]), while the lowest rated was item 20 ‘the potential resource implications of applying the recommendations have been considered’ (domain 5) (1 [1–1]). Raw consensus item scores are available in Table S4. Only three guidelines scored ≥70% for domain 3 ‘rigour of development’, proceeding to AGREE-REX appraisal.

**Table 2:** AGREE-II Quality Assessment of Included Guidelines (*n* = 15).

Colour Legend:  < 25%,  25% − 49%,  50% − 74%,  ≥75%

Domains: 1 – Scope and Purpose; 2 – Stakeholder Involvement; 3 – Rigour of Development; 4 – Clarity of Presentation; 5 – Applicability; 6 – Editorial Independence.

*AGREE-II* Appraisal of Guidelines Research and Evaluation, Version II, *AIOSS* Italian Association of Stoma care Nurses, *ANIGEA* Italian Association of Gastroenterology Nurses and Associates, *ANOTE* Italian Association of Endoscopic Operators, *ASPEN* American Society for Parenteral and Enteral Nutrition, *BSG* British Society of Gastroenterology, *CREST* Clinical Resource Efficiency Support Team, *ECFS* European Cystic Fibrosis Society, *ESGE* European Society of Gastrointestinal Endoscopy, *ESPEN* European Society for Clinical Nutrition and Metabolism, *GENCA* Gastroenterological Nurses College of Australia, *HAS* Haute Autorité de santé (French National Authority for Health), *KSGE* Korean Society of Gastrointestinal Endoscopy, *NICE* National Institute for Health and Care Excellence, *NSW ACI* New South Wales Agency for Clinical Innovation, *IQR* Interquartile Range.

### Guideline recommendations and their assessment using the AGREE-REX tool

Of all recommendations 66% (72/109) were graded as Good Practice Points while 6% (6/109) were of grade A, 18% (20/109) of grade B and 10% (11/109) of grade O. All recommendations were grouped into six clusters which were each assessed using the AGREE-REX tool. A summary of recommendation grades stratified by cluster and guideline is presented in Table S5.

Clusters for all guidelines scored below 70% for all three domains of the AGREE-REX tool, with no overall score for any cluster >50% (Table 3). Across all guidelines, ‘Clinical Applicability’ (56% [39–61%]) was the highest scoring domain, with ‘care of feeding sites and enteral access devices’ (61% [33–61%]) and ‘recommendations for HETF feeds’ (61% [56–67%]) the highest scoring clusters in this domain. These clusters also consisted of the highest proportion of A-O graded recommendations with 45% (17/38) and 45% (9/20) respectively (Table S5). ‘Values and Preferences’ (21% [17–25%]) was the lowest scoring domain with the corresponding item 6 ‘values and preferences of policy/decision-makers’ (1[1–2]) and item 7 ‘values and preferences of guideline developers’ (1 [1–2]) the lowest scoring items. Raw consensus item scores are available in Table S6. All recommendation clusters were recommended for use with modifications (Table S6).

**Table 3:** AGREE-REX Quality Assessment of Six Recommendation Clusters from Included Guidelines (*n* = 3).

Colour Legend:  < 25%,  25% − 49%,  50% − 74%, nil values ≥ 75%

^a^Domains: 1 – Clinical Applicability; 2 - Values and Preferences; 3 - Implementability.

^b^Recommendation Clusters: A- Commencement of HETF; B - Care of Feeding Site and Enteral Access Devices; C - Recommendations for Feeding; D - Monitoring HETF; E - Termination of HETF; F - Requirements for HETF Services.

*ESPEN* European Society for Clinical Nutrition and Metabolism, *NICE* National Institute for Health and Care Excellence, *IQR* Interquartile Range.

## Discussion

This review is the first to systematically identify, synthesise and evaluate the methodological quality of available guidelines and their recommendations for HETF. We identified and assessed 15 guidelines of which many were of poor methodological quality, either failing to employ systematic searches of the literature, utilising informal consensus methods, or simply lacking transparency and detail as to the methods employed. Only three of the identified guidelines were of high methodological quality however none of the recommendation clusters relevant to HETF from these guidelines achieved an overall AGREE-REX quality score > 50%. Numerous low-scoring items and domains from both the AGREE-II and AGREE-REX evaluations were attributed to the same methodological shortcomings of the appraised guidelines.

‘Rigour of development’ has been acknowledged as a critical domain in the appraisal of guideline quality [42]. While scores in this domain were low there was an improvement in the use of systematic search methods and structured consensus over time. A noticeable limitation of most guidelines was the absence of criteria for regular update with only four guidelines ever having been renewed. In the past six years, no updates had been made. ‘Evidence’ scores were generally low with a majority of recommendations being good practice points, based on low-level evidence or extrapolated from moderate-level evidence. Inconsistent alignment between the definitiveness of some of these good practice point recommendations and the rationale provided, or lack thereof, was the main reason for lower scoring on ‘applicability to target users’. Discordant recommendations, that is, when the strength of a recommendation is not reflective of the certainty of evidence, are warranted when there is low quality evidence suggesting benefit in life-threatening situations [43–46]. The absence of clinical equipoise in providing EN would constitute the conduct of certain randomised controlled trials in this population unethical [47]. When evidence is limited, guideline developers should be transparent as to the considerations made when formulating recommendations. This would include the perspective taken, the value assigned to relevant outcomes, acceptability of the recommendation to relevant stakeholders, resource requirements and the feasibility of implementation [48, 49]. Scarcity of high-quality evidence should not deter guideline developers from creating or updating clinical guidance, rather the well documented limitations of the evidence base could support a redistribution of some resources to rigorous conduct in other aspects of guideline development such as stakeholder engagement and ensuring implementability. Benchmarking studies of local guideline uptake have demonstrated inconsistent adherence [7] and thus further engagement with HETF stakeholders including people receiving HETF, their carers, clinicians involved in service provision, service managers and guideline and policy-makers, would be warranted.

The practical utility of a guideline is only possible when developed with a plan of implementation. Most guidelines disregarded facilitators, barriers, and resource considerations to applying their recommendations. Qualitative studies with HETF service users and providers have highlighted the benefit of having access to providers with expertise in HETF including management of complications [50]. There are often financial barriers however in accessing services and challenges with co-ordinating care by a multidisciplinary team [10]. Oftentimes what may be clinically most suitable for a patient (e.g. feeding mode, formula choice or frequency of review) may not be feasible within the client and health service resources. Guidelines acknowledged implementation barriers and facilitators although tools and resources to address these were infrequently provided as were monitoring and auditing criteria. This may in part be secondary to the minimal involvement of key stakeholders in the development process and external review of these guidelines. Quality appraisals of guidelines for critical care nutrition and medical nutrition therapy in liver cirrhosis have also highlighted similar limitations in stakeholder consultation, applicability and consideration of values and preferences in formulating recommendations [51–54]. Economic analyses are also crucial in assuring the implementability of recommendations [55] although were noticeably absent in the guidelines assessed. Cost savings have been reported with the implementation of HETF-dedicated outpatient clinics [56] and advanced practice dietitian roles in gastrostomy management [57] – both practices which would have allowed for closer adherence to HETF guidelines. With ethical restrictions in the conduct of RCTs, economic modelling may serve as a more suitable approach to showcase the benefit of recommendations made for this population group.

All three high quality guidelines were recommended for use with modifications secondary to the prospect of improving quality with subsequent updates. NICE guidance was originally developed over two decades ago with an overall high methodological quality although with some informal consensus methods. New evidence has been considered in the need to update this guideline [58] and some supporting tools to facilitate implementation have been provided [59, 60] although the healthcare space has changed drastically since its original inception. For instance, community-based replacement of gastrostomy tubes following traumatic displacement has become more commonplace following the COVID-19 pandemic [61] with the NHS also issuing a recovery plan for urgent and emergency services which involved expanded community services [62]. The guideline would now likely benefit from more current and meaningful consultation with stakeholders to ensure its relevance and usability [63]. Similarly, ESPEN guidelines’ quality could also be improved through wider or more clearly explicated consultation with consumers and policymakers. This has proven difficult secondary to unclear guidance [64] although some direction has been provided and the Cochrane Multi-Stakeholder Engagement (MuSE) Consortium seeks to provide guidance on the matter [65].

All guidelines were developed by professional associations and/or government agencies with suboptimal reporting of editorial independence. Funding body influence was not always applicable as many professional bodies are interested in generating guidelines to improve patient management, thus assigned scores of ‘1 (absence of information)’ reduced the overall domain 6 score. Conflict of interest was however poorly reported with most guidelines not describing the type of competing interests considered nor the methods employed in attaining them. While it is likely editorial independence existed for most guidelines, many did not indicate this correctly, similarly to other aspects of guideline development.

### Strengths and limitations

Strengths of this review include using a systematic search of five databases and an extensive grey literature search where authors were contacted to source supporting materials. This study also used the AGREE-II and AGREE-REX tools which are reliable and valid tools dedicated to assessing the quality of guidelines and their recommendations. While the minimum number of appraisers assessed each guideline, a consensus method was used in both assessments to address this. Finally, predominantly publicly available guidelines were included, and it is possible that documents produced by associations only accessible via subscription were missed.

## Conclusions

This review identified and analysed 15 guidelines regarding home enteral tube feeding from seven geographical regions, three of which were considered high quality. Despite their higher quality, even the NICE and ESPEN guidelines had methodological weaknesses which limit their usability and for this reason no guideline or recommendation cluster was recommended for use without modification. These findings highlight the importance of transparent and detailed reporting practices and the need to consider meaningful involvement of people receiving HETF and their carers, clinicians and service managers in guideline development to ensure recommendation applicability and implementability. Guideline developers should invest in economic analyses to accompany recommendations relating to service structure and provide targeted tools and resources such as competency criteria to support education and training of both clinicians and people receiving HETF.

## Supplementary information

Supplementary Material _Table S1

Supplementary Material_Table S2-S6
