# Supplementary material for: A systematic review and quality appraisal of guidelines and recommendations for home enteral tube feeding in adults
Source: Eur J Clin Nutr. 2024 Sep 3;79(2):104–12. doi: 10.1038/s41430-024-01500-1 (PMC11810790; doi:10.1038/s41430-024-01500-1)
Supplement: Supplementary file 1 — Supplementary Material _Table S1 [file 41430_2024_1500_MOESM1_ESM.docx]

## Supplementary Materials

**Table S1. Search Strategies**

| **Medline via OVID** | |
| --- | --- |
| 1 | duodenostomy/ |
| 2 | Enteral Nutrition/ |
| 3 | enterostomy/ |
| 4 | gastrostomy/ |
| 5 | Intubation, Gastrointestinal/ |
| 6 | jejunostomy/ |
| 7 | nutrition therapy/ |
| 8 | nutritional support/ |
| 9 | ((artificial* adj2 nutrition) or (artificial* adj2 fed) or (artificial* adj2 feed*) or (artificial* adj2 therap*) or (artificial* adj2 support*) or (artificial* adj2 diet*)).tw,kf. |
| 10 | ((duoden* adj2 nutrition) or (duoden* adj2 fed) or (duoden* adj2 feed*) or (duoden* adj2 therap*) or (duoden* adj2 support*) or (duoden* adj2 diet*)).tw,kf. |
| 11 | ((enter* adj2 nutrition) or (enter* adj2 fed) or (enter* adj2 feed*) or (enter* adj2 therap*) or (enter* adj2 support*) or (enter* adj2 diet*)).tw,kf. |
| 12 | ((gastr* adj2 nutrition) or (gastr* adj2 fed) or (gastr* adj2 feed*) or (gastr* adj2 therap*) or (gastr* adj2 support*) or (gastr* adj2 diet*)).tw,kf. |
| 13 | ((intestinal* adj2 nutrition) or (intestinal* adj2 fed) or (intestinal* adj2 feed*) or (intestinal* adj2 therap*) or (intestinal* adj2 support*) or (intestinal* adj2 diet*)).tw,kf. |
| 14 | ((intragastr* adj2 nutrition) or (intragastr* adj2 fed) or (intragastr* adj2 feed*) or (intragastr* adj2 therap*) or (intragastr* adj2 support*) or (intragastr* adj2 diet*)).tw,kf. |
| 15 | ((jejun* adj2 nutrition) or (jejun* adj2 fed) or (jejun* adj2 feed*) or (jejun* adj2 therap*) or (jejun* adj2 support*) or (jejun* adj2 diet*)).tw,kf. |
| 16 | ((nasogastric* adj2 nutrition) or (nasogastric* adj2 fed) or (nasogastric* adj2 feed*) or (nasogastric* adj2 therap*) or (nasogastric* adj2 support*) or (nasogastric* adj2 diet*) or (naso-gastric* adj2 nutrition) or (naso-gastric* adj2 fed) or (naso-gastric* adj2 feed*) or (naso-gastric* adj2 therap*) or (naso-gastric* adj2 support*) or (naso-gastric* adj2 diet*) or (nasojejunal* adj2 nutrition) or (nasojejunal* adj2 fed) or (nasojejunal* adj2 feed*) or (nasojejunal* adj2 therap*) or (nasojejunal* adj2 support*) or (nasojejunal* adj2 diet*) or (naso-jejunal* adj2 nutrition) or (naso-jejunal* adj2 fed) or (naso-jejunal* adj2 feed*) or (naso-jejunal* adj2 therap*) or (naso-jejunal* adj2 support*) or (naso-jejunal* adj2 diet*)).tw,kf. |
| 17 | ((ngt adj2 nutrition) or (ngt adj2 fed) or (ngt adj2 feed*) or (ngt adj2 therap*) or (ngt adj2 support*) or (ngt adj2 diet*)).tw,kf. |
| 18 | ((njt adj2 nutrition) or (njt adj2 fed) or (njt adj2 feed*) or (njt adj2 therap*) or (njt adj2 support*) or (njt adj2 diet*)).tw,kf. |
| 19 | ((non oral adj2 nutrition) or (non oral adj2 fed) or (non oral adj2 feed*) or (non oral adj2 therap*) or (non oral adj2 support*) or (non oral adj2 diet*)).tw,kf. |
| 20 | ((nonoral adj2 nutrition) or (nonoral adj2 fed) or (nonoral adj2 feed*) or (nonoral adj2 therap*) or (nonoral adj2 support*) or (nonoral adj2 diet*)).tw,kf. |
| 21 | ((orogastric* adj2 nutrition) or (orogastric* adj2 fed) or (orogastric* adj2 feed*) or (orogastric* adj2 therap*) or (orogastric* adj2 support*) or (orogastric* adj2 diet*) or (oro-gastric* adj2 nutrition) or (oro-gastric* adj2 fed) or (oro-gastric* adj2 feed*) or (oro-gastric* adj2 therap*) or (oro-gastric* adj2 support*) or (oro-gastric* adj2 diet*) or (orojejunal* adj2 nutrition) or (orojejunal* adj2 fed) or (orojejunal* adj2 feed*) or (orojejunal* adj2 therap*) or (orojejunal* adj2 support*) or (orojejunal* adj2 diet*) or (oro-jejunal* adj2 nutrition) or (oro-jejunal* adj2 fed) or (oro-jejunal* adj2 feed*) or (oro-jejunal* adj2 therap*) or (oro-jejunal* adj2 support*) or (oro-jejunal* adj2 diet*)).tw,kf. |
| 22 | ((stomach adj2 nutrition) or (stomach adj2 fed) or (stomach adj2 feed*) or (stomach adj2 therap*) or (stomach adj2 support*) or (stomach adj2 diet*)).tw,kf. |
| 23 | ((tube adj2 nutrition) or (tube adj2 fed) or (tube adj2 feed*) or (tube adj2 therap*) or (tube adj2 support*) or (tube adj2 diet*)).tw,kf. |
| 24 | 1 or 2 or 3 or 4 or 5 or 6 or 7 or 8 or 9 or 10 or 11 or 12 or 13 or 14 or 15 or 16 or 17 or 18 or 19 or 20 or 21 or 22 or 23 |
| 25 | adult day care centers/ |
| 26 | community health services/ |
| 27 | community networks/ |
| 28 | exp community health nursing/ |
| 29 | exp dietary services/ |
| 30 | exp home nursing/ |
| 31 | home care services/ |
| 32 | home care services, hospital-based/ |
| 33 | hospices/ |
| 34 | 25 or 26 or 27 or 28 or 29 or 30 or 31 or 32 or 33 |
| 35 | 24 and 34 |
| 36 | ((gastrostomy adj2 nutrition) or (gastrostomy adj2 fed) or (gastrostomy adj2 feed*) or (gastrostomy adj2 therap*) or (gastrostomy adj2 support*) or (gastrostomy adj2 diet*)).tw,kf. |
| 37 | ((jejunostomy adj2 nutrition) or (jejunostomy adj2 fed) or (jejunostomy adj2 feed*) or (jejunostomy adj2 therap*) or (jejunostomy adj2 support*) or (jejunostomy adj2 diet*)).tw,kf. |
| 38 | ((peg j adj2 nutrition) or (peg j adj2 fed) or (peg j adj2 feed*) or (peg j adj2 therap*) or (peg j adj2 support*) or (peg j adj2 diet*)).tw,kf. |
| 39 | ((pegj adj2 nutrition) or (pegj adj2 fed) or (pegj adj2 feed*) or (pegj adj2 therap*) or (pegj adj2 support*) or (pegj adj2 diet*)).tw,kf. |
| 40 | ((pej adj2 nutrition) or (pej adj2 fed) or (pej adj2 feed*) or (pej adj2 therap*) or (pej adj2 support*) or (pej adj2 diet*)).tw,kf. |
| 41 | ((percutaneous endoscopic gastrostomy adj2 nutrition) or (percutaneous endoscopic gastrostomy adj2 fed) or (percutaneous endoscopic gastrostomy adj2 feed*) or (percutaneous endoscopic gastrostomy adj2 therap*) or (percutaneous endoscopic gastrostomy adj2 support*) or (percutaneous endoscopic gastrostomy adj2 diet*)).tw,kf. |
| 42 | ((percutaneous endoscopic jejunostomy adj2 nutrition) or (percutaneous endoscopic jejunostomy adj2 fed) or (percutaneous endoscopic jejunostomy adj2 feed*) or (percutaneous endoscopic jejunostomy adj2 therap*) or (percutaneous endoscopic jejunostomy adj2 support*) or (percutaneous endoscopic jejunostomy adj2 diet*)).tw,kf. |
| 43 | ((radiologically inserted gastrostomy adj2 nutrition) or (radiologically inserted gastrostomy adj2 fed) or (radiologically inserted gastrostomy adj2 feed*) or (radiologically inserted gastrostomy adj2 therap*) or (radiologically inserted gastrostomy adj2 support*) or (radiologically inserted gastrostomy adj2 diet*)).tw,kf. |
| 44 | 36 or 37 or 38 or 39 or 40 or 41 or 42 or 43 |
| 45 | ((home or communit* or long term or longterm or residential* or outpatient or lifelong or life long or ambulatory) adj3 ((artificial* adj2 nutrition) or (artificial* adj2 fed) or (artificial* adj2 feed*) or (artificial* adj2 therap*) or (artificial* adj2 support*) or (artificial* adj2 diet*) or ((duoden* adj2 nutrition) or (duoden* adj2 fed) or (duoden* adj2 feed*) or (duoden* adj2 therap*) or (duoden* adj2 support*) or (duoden* adj2 diet*)) or ((enter* adj2 nutrition) or (enter* adj2 fed) or (enter* adj2 feed*) or (enter* adj2 therap*) or (enter* adj2 support*) or (enter* adj2 diet*)) or ((gastr* adj2 nutrition) or (gastr* adj2 fed) or (gastr* adj2 feed*) or (gastr* adj2 therap*) or (gastr* adj2 support*) or (gastr* adj2 diet*)) or ((intestinal* adj2 nutrition) or (intestinal* adj2 fed) or (intestinal* adj2 feed*) or (intestinal* adj2 therap*) or (intestinal* adj2 support*) or (intestinal* adj2 diet*)) or ((intragastr* adj2 nutrition) or (intragastr* adj2 fed) or (intragastr* adj2 feed*) or (intragastr* adj2 therap*) or (intragastr* adj2 support*) or (intragastr* adj2 diet*)) or ((jejun* adj2 nutrition) or (jejun* adj2 fed) or (jejun* adj2 feed*) or (jejun* adj2 therap*) or (jejun* adj2 support*) or (jejun* adj2 diet*)) or ((naso* adj2 nutrition) or (naso* adj2 fed) or (naso* adj2 feed*) or (naso* adj2 therap*) or (naso* adj2 support*) or (naso* adj2 diet*)) or ((ngt adj2 nutrition) or (ngt adj2 fed) or (ngt adj2 feed*) or (ngt adj2 therap*) or (ngt adj2 support*) or (ngt adj2 diet*)) or ((njt adj2 nutrition) or (njt adj2 fed) or (njt adj2 feed*) or (njt adj2 therap*) or (njt adj2 support*) or (njt adj2 diet*)) or ((non oral adj2 nutrition) or (non oral adj2 fed) or (non oral adj2 feed*) or (non oral adj2 therap*) or (non oral adj2 support*) or (non oral adj2 diet*)) or ((nonoral adj2 nutrition) or (nonoral adj2 fed) or (nonoral adj2 feed*) or (nonoral adj2 therap*) or (nonoral adj2 support*) or (nonoral adj2 diet*)) or ((oro* adj2 nutrition) or (oro* adj2 fed) or (oro* adj2 feed*) or (oro* adj2 therap*) or (oro* adj2 support*) or (oro* adj2 diet*)) or ((stomach adj2 nutrition) or (stomach adj2 fed) or (stomach adj2 feed*) or (stomach adj2 therap*) or (stomach adj2 support*) or (stomach adj2 diet*)) or ((tube adj2 nutrition) or (tube adj2 fed) or (tube adj2 feed*) or (tube adj2 therap*) or (tube adj2 support*) or (tube adj2 diet*)))).tw,kf. |
| 46 | 35 or 44 or 45 |
| 47 | exp adult/ or exp Geriatrics/ or (adult* or m#n or wom#n or elder* or senior* or (old* adj (person* or people* or population* or patient* or subject* or male* or female*))).tw,kf. |
| 48 | 46 and 47 |
| 49 | Guideline/ |
| 50 | Clinical Protocols/ |
| 51 | Consensus/ |
| 52 | (CPG* or guideline* or guidance or guide* or consensus or polic* or pathway* or recommendation* or regulation* or standard* or summar* or statement*).tw,kf. |
| 53 | ((position* adj2 paper) or (position* adj2 stand)).tw,kf. |
| 54 | (practice adj3 parameter*).tw,kf. |
| 55 | health policy/ or health care reform/ or nutrition policy/ |
| 56 | exp Consensus Development Conference/ |
| 57 | health planning/ or health planning guidelines/ |
| 58 | exp Guideline/ |
| 59 | 49 or 50 or 51 or 52 or 53 or 54 or 55 or 56 or 57 or 58 |
| 60 | 48 and 59 |
| 61 | limit 60 to yr="2000 -Current" |
| **Embase via OVID** | |
| 1 | artificial feeding/ |
| 2 | digestive catheter/ |
| 3 | digestive tract intubation/ |
| 4 | digestive tube/ |
| 5 | duodenum intubation/ |
| 6 | enteric feeding/ |
| 7 | enterostomy tube/ |
| 8 | feeding tube/ |
| 9 | gastrointestinal intubation tube/ |
| 10 | gastrojejunostomy tube/ |
| 11 | gastrostoma/ |
| 12 | gastrostomy button/ |
| 13 | gastrostomy catheter/ |
| 14 | gastrostomy device/ |
| 15 | jejunostomy catheter/ |
| 16 | jejunostomy tube/ |
| 17 | levine tube/ |
| 18 | nasogastric feeding apparatus/ |
| 19 | nasogastric tube/ |
| 20 | nose feeding/ |
| 21 | orogastric tube/ |
| 22 | ostomy catheter/ |
| 23 | ostomy tube/ |
| 24 | percutaneous endoscopic gastrostomy tube/ |
| 25 | postpyloric feeding tube/ |
| 26 | small-bore nasogastric tube/ |
| 27 | stomach intubation/ |
| 28 | stomach tube/ |
| 29 | ((artificial* adj2 nutrition) or (artificial* adj2 fed) or (artificial* adj2 feed*) or (artificial* adj2 therap*) or (artificial* adj2 support*) or (artificial* adj2 diet*)).tw,kf. |
| 30 | ((duoden* adj2 nutrition) or (duoden* adj2 fed) or (duoden* adj2 feed*) or (duoden* adj2 therap*) or (duoden* adj2 support*) or (duoden* adj2 diet*)).tw,kf. |
| 31 | ((enter* adj2 nutrition) or (enter* adj2 fed) or (enter* adj2 feed*) or (enter* adj2 therap*) or (enter* adj2 support*) or (enter* adj2 diet*)).tw,kf. |
| 32 | ((gastr* adj2 nutrition) or (gastr* adj2 fed) or (gastr* adj2 feed*) or (gastr* adj2 therap*) or (gastr* adj2 support*) or (gastr* adj2 diet*)).tw,kf. |
| 33 | ((intestinal* adj2 nutrition) or (intestinal* adj2 fed) or (intestinal* adj2 feed*) or (intestinal* adj2 therap*) or (intestinal* adj2 support*) or (intestinal* adj2 diet*)).tw,kf. |
| 34 | ((intragastr* adj2 nutrition) or (intragastr* adj2 fed) or (intragastr* adj2 feed*) or (intragastr* adj2 therap*) or (intragastr* adj2 support*) or (intragastr* adj2 diet*)).tw,kf. |
| 35 | ((jejun* adj2 nutrition) or (jejun* adj2 fed) or (jejun* adj2 feed*) or (jejun* adj2 therap*) or (jejun* adj2 support*) or (jejun* adj2 diet*)).tw,kf. |
| 36 | ((nasogastric* adj2 nutrition) or (nasogastric* adj2 fed) or (nasogastric* adj2 feed*) or (nasogastric* adj2 therap*) or (nasogastric* adj2 support*) or (nasogastric* adj2 diet*) or (naso-gastric* adj2 nutrition) or (naso-gastric* adj2 fed) or (naso-gastric* adj2 feed*) or (naso-gastric* adj2 therap*) or (naso-gastric* adj2 support*) or (naso-gastric* adj2 diet*) or (nasojejunal* adj2 nutrition) or (nasojejunal* adj2 fed) or (nasojejunal* adj2 feed*) or (nasojejunal* adj2 therap*) or (nasojejunal* adj2 support*) or (nasojejunal* adj2 diet*) or (naso-jejunal* adj2 nutrition) or (naso-jejunal* adj2 fed) or (naso-jejunal* adj2 feed*) or (naso-jejunal* adj2 therap*) or (naso-jejunal* adj2 support*) or (naso-jejunal* adj2 diet*)).tw,kf. |
| 37 | ((ngt adj2 nutrition) or (ngt adj2 fed) or (ngt adj2 feed*) or (ngt adj2 therap*) or (ngt adj2 support*) or (ngt adj2 diet*)).tw,kf. |
| 38 | ((njt adj2 nutrition) or (njt adj2 fed) or (njt adj2 feed*) or (njt adj2 therap*) or (njt adj2 support*) or (njt adj2 diet*)).tw,kf. |
| 39 | ((non oral adj2 nutrition) or (non oral adj2 fed) or (non oral adj2 feed*) or (non oral adj2 therap*) or (non oral adj2 support*) or (non oral adj2 diet*)).tw,kf. |
| 40 | ((nonoral adj2 nutrition) or (nonoral adj2 fed) or (nonoral adj2 feed*) or (nonoral adj2 therap*) or (nonoral adj2 support*) or (nonoral adj2 diet*)).tw,kf. |
| 41 | ((orogastric* adj2 nutrition) or (orogastric* adj2 fed) or (orogastric* adj2 feed*) or (orogastric* adj2 therap*) or (orogastric* adj2 support*) or (orogastric* adj2 diet*) or (oro-gastric* adj2 nutrition) or (oro-gastric* adj2 fed) or (oro-gastric* adj2 feed*) or (oro-gastric* adj2 therap*) or (oro-gastric* adj2 support*) or (oro-gastric* adj2 diet*) or (orojejunal* adj2 nutrition) or (orojejunal* adj2 fed) or (orojejunal* adj2 feed*) or (orojejunal* adj2 therap*) or (orojejunal* adj2 support*) or (orojejunal* adj2 diet*) or (oro-jejunal* adj2 nutrition) or (oro-jejunal* adj2 fed) or (oro-jejunal* adj2 feed*) or (oro-jejunal* adj2 therap*) or (oro-jejunal* adj2 support*) or (oro-jejunal* adj2 diet*)).tw,kf. |
| 42 | ((stomach adj2 nutrition) or (stomach adj2 fed) or (stomach adj2 feed*) or (stomach adj2 therap*) or (stomach adj2 support*) or (stomach adj2 diet*)).tw,kf. |
| 43 | ((tube adj2 nutrition) or (tube adj2 fed) or (tube adj2 feed*) or (tube adj2 therap*) or (tube adj2 support*) or (tube adj2 diet*)).tw,kf. |
| 44 | 1 or 2 or 3 or 4 or 5 or 6 or 7 or 8 or 9 or 10 or 11 or 12 or 13 or 14 or 15 or 16 or 17 or 18 or 19 or 20 or 21 or 22 or 23 or 24 or 25 or 26 or 27 or 28 or 29 or 30 or 31 or 32 or 33 or 34 or 35 or 36 or 37 or 38 or 39 or 40 or 41 or 42 or 43 |
| 45 | community based rehabilitation/ |
| 46 | community based surveillance/ |
| 47 | community care/ |
| 48 | community health nursing/ |
| 49 | community integration/ |
| 50 | community program/ |
| 51 | community support/ |
| 52 | dietetics/ |
| 53 | exp ambulatory care/ |
| 54 | exp secondary health care/ |
| 55 | home care/ |
| 56 | home for the aged/ |
| 57 | home health agency/ |
| 58 | home monitoring/ |
| 59 | home rehabilitation/ |
| 60 | home visit/ |
| 61 | institutional care/ |
| 62 | long term care/ |
| 63 | neurorehabilitation/ |
| 64 | nutrition service/ |
| 65 | nutritional support/ |
| 66 | residential care/ |
| 67 | rehabilitation/ |
| 68 | rehabilitation care/ |
| 69 | residential care/ |
| 70 | respite care/ |
| 71 | stroke rehabilitation/ |
| 72 | visiting nursing service/ |
| 73 | 45 or 46 or 47 or 48 or 49 or 50 or 51 or 52 or 53 or 54 or 55 or 56 or 57 or 58 or 59 or 60 or 61 or 62 or 63 or 64 or 65 or 66 or 67 or 68 or 69 or 70 or 71 or 72 |
| 74 | 44 and 73 |
| 75 | ((gastrostomy adj2 nutrition) or (gastrostomy adj2 fed) or (gastrostomy adj2 feed*) or (gastrostomy adj2 therap*) or (gastrostomy adj2 support*) or (gastrostomy adj2 diet*)).tw,kf. |
| 76 | ((jejunostomy adj2 nutrition) or (jejunostomy adj2 fed) or (jejunostomy adj2 feed*) or (jejunostomy adj2 therap*) or (jejunostomy adj2 support*) or (jejunostomy adj2 diet*)).tw,kf. |
| 77 | ((peg j adj2 nutrition) or (peg j adj2 fed) or (peg j adj2 feed*) or (peg j adj2 therap*) or (peg j adj2 support*) or (peg j adj2 diet*)).tw,kf. |
| 78 | ((pegj adj2 nutrition) or (pegj adj2 fed) or (pegj adj2 feed*) or (pegj adj2 therap*) or (pegj adj2 support*) or (pegj adj2 diet*)).tw,kf. |
| 79 | ((pej adj2 nutrition) or (pej adj2 fed) or (pej adj2 feed*) or (pej adj2 therap*) or (pej adj2 support*) or (pej adj2 diet*)).tw,kf. |
| 80 | ((percutaneous endoscopic gastrostomy adj2 nutrition) or (percutaneous endoscopic gastrostomy adj2 fed) or (percutaneous endoscopic gastrostomy adj2 feed*) or (percutaneous endoscopic gastrostomy adj2 therap*) or (percutaneous endoscopic gastrostomy adj2 support*) or (percutaneous endoscopic gastrostomy adj2 diet*)).tw,kf. |
| 81 | ((percutaneous endoscopic jejunostomy adj2 nutrition) or (percutaneous endoscopic jejunostomy adj2 fed) or (percutaneous endoscopic jejunostomy adj2 feed*) or (percutaneous endoscopic jejunostomy adj2 therap*) or (percutaneous endoscopic jejunostomy adj2 support*) or (percutaneous endoscopic jejunostomy adj2 diet*)).tw,kf. |
| 82 | ((radiologically inserted gastrostomy adj2 nutrition) or (radiologically inserted gastrostomy adj2 fed) or (radiologically inserted gastrostomy adj2 feed*) or (radiologically inserted gastrostomy adj2 therap*) or (radiologically inserted gastrostomy adj2 support*) or (radiologically inserted gastrostomy adj2 diet*)).tw,kf. |
| 83 | 75 or 76 or 77 or 78 or 79 or 80 or 81 or 82 |
| 84 | ((home or communit* or long term or longterm or residential* or outpatient or lifelong or life long or ambulatory) adj3 ((artificial* adj2 nutrition) or (artificial* adj2 fed) or (artificial* adj2 feed*) or (artificial* adj2 therap*) or (artificial* adj2 support*) or (artificial* adj2 diet*) or ((duoden* adj2 nutrition) or (duoden* adj2 fed) or (duoden* adj2 feed*) or (duoden* adj2 therap*) or (duoden* adj2 support*) or (duoden* adj2 diet*)) or ((enter* adj2 nutrition) or (enter* adj2 fed) or (enter* adj2 feed*) or (enter* adj2 therap*) or (enter* adj2 support*) or (enter* adj2 diet*)) or ((gastr* adj2 nutrition) or (gastr* adj2 fed) or (gastr* adj2 feed*) or (gastr* adj2 therap*) or (gastr* adj2 support*) or (gastr* adj2 diet*)) or ((intestinal* adj2 nutrition) or (intestinal* adj2 fed) or (intestinal* adj2 feed*) or (intestinal* adj2 therap*) or (intestinal* adj2 support*) or (intestinal* adj2 diet*)) or ((intragastr* adj2 nutrition) or (intragastr* adj2 fed) or (intragastr* adj2 feed*) or (intragastr* adj2 therap*) or (intragastr* adj2 support*) or (intragastr* adj2 diet*)) or ((jejun* adj2 nutrition) or (jejun* adj2 fed) or (jejun* adj2 feed*) or (jejun* adj2 therap*) or (jejun* adj2 support*) or (jejun* adj2 diet*)) or ((naso* adj2 nutrition) or (naso* adj2 fed) or (naso* adj2 feed*) or (naso* adj2 therap*) or (naso* adj2 support*) or (naso* adj2 diet*)) or ((ngt adj2 nutrition) or (ngt adj2 fed) or (ngt adj2 feed*) or (ngt adj2 therap*) or (ngt adj2 support*) or (ngt adj2 diet*)) or ((njt adj2 nutrition) or (njt adj2 fed) or (njt adj2 feed*) or (njt adj2 therap*) or (njt adj2 support*) or (njt adj2 diet*)) or ((non oral adj2 nutrition) or (non oral adj2 fed) or (non oral adj2 feed*) or (non oral adj2 therap*) or (non oral adj2 support*) or (non oral adj2 diet*)) or ((nonoral adj2 nutrition) or (nonoral adj2 fed) or (nonoral adj2 feed*) or (nonoral adj2 therap*) or (nonoral adj2 support*) or (nonoral adj2 diet*)) or ((oro* adj2 nutrition) or (oro* adj2 fed) or (oro* adj2 feed*) or (oro* adj2 therap*) or (oro* adj2 support*) or (oro* adj2 diet*)) or ((stomach adj2 nutrition) or (stomach adj2 fed) or (stomach adj2 feed*) or (stomach adj2 therap*) or (stomach adj2 support*) or (stomach adj2 diet*)) or ((tube adj2 nutrition) or (tube adj2 fed) or (tube adj2 feed*) or (tube adj2 therap*) or (tube adj2 support*) or (tube adj2 diet*)))).tw,kf. |
| 85 | 74 or 83 or 84 |
| 86 | adult/ or institutionalized adult/ or middle aged/ or young adult/ or aged/ or frail elderly/ or institutionalized elderly/ or very elderly/ or geriatrics/ or (adult* or m#n or wom#n or elder* or senior* or (old* adj (person* or people* or female* or male* or population* or subject* or patient*))).tw,kf. |
| 87 | 85 and 86 |
| 88 | health care planning/ |
| 89 | hospital policy/ |
| 90 | (practice adj3 parameter*).tw,kf. |
| 91 | ((position* adj2 paper) or (position* adj2 stand)).tw,kf. |
| 92 | (CPG* or guideline* or guidance or guide* or consensus or polic* or pathway* or recommendation* or regulation* or standard* or summar* or statement*).tw,kf. |
| 93 | professional standard/ or consensus/ |
| 94 | practice guideline/ or clinical pathway/ or consensus development/ or good clinical practice/ |
| 95 | exp health care policy/ |
| 96 | ((healthcare adj2 plan*) or (health care adj2 plan) or (treat* adj2 plan*)).tw,kf. |
| 97 | 88 or 89 or 90 or 91 or 92 or 93 or 94 or 95 or 96 or 97 |
| 98 | 87 and 97 |
| 99 | limit 98 to yr="2000 -Current" |
| **PsycINFO via OVID** | |
| 1 | community services/ |
| 2 | exp outpatient treatment/ |
| 3 | home care/ |
| 4 | home visiting programs/ |
| 5 | long term care/ |
| 6 | 1 or 2 or 3 or 4 or 5 |
| 7 | ((artificial* adj2 nutrition) or (artificial* adj2 fed) or (artificial* adj2 feed*) or (artificial* adj2 therap*) or (artificial* adj2 support*) or (artificial* adj2 diet*)).tw,id. |
| 8 | ((duoden* adj2 nutrition) or (duoden* adj2 fed) or (duoden* adj2 feed*) or (duoden* adj2 therap*) or (duoden* adj2 support*) or (duoden* adj2 diet*)).tw,id. |
| 9 | ((enter* adj2 nutrition) or (enter* adj2 fed) or (enter* adj2 feed*) or (enter* adj2 therap*) or (enter* adj2 support*) or (enter* adj2 diet*)).tw,id. |
| 10 | ((gastr* adj2 nutrition) or (gastr* adj2 fed) or (gastr* adj2 feed*) or (gastr* adj2 therap*) or (gastr* adj2 support*) or (gastr* adj2 diet*)).tw,id. |
| 11 | ((intestinal* adj2 nutrition) or (intestinal* adj2 fed) or (intestinal* adj2 feed*) or (intestinal* adj2 therap*) or (intestinal* adj2 support*) or (intestinal* adj2 diet*)).tw,id. |
| 12 | ((intragastr* adj2 nutrition) or (intragastr* adj2 fed) or (intragastr* adj2 feed*) or (intragastr* adj2 therap*) or (intragastr* adj2 support*) or (intragastr* adj2 diet*)).tw,id. |
| 13 | ((jejun* adj2 nutrition) or (jejun* adj2 fed) or (jejun* adj2 feed*) or (jejun* adj2 therap*) or (jejun* adj2 support*) or (jejun* adj2 diet*)).tw,id. |
| 14 | ((nasogastric* adj2 nutrition) or (nasogastric* adj2 fed) or (nasogastric* adj2 feed*) or (nasogastric* adj2 therap*) or (nasogastric* adj2 support*) or (nasogastric* adj2 diet*) or (naso-gastric* adj2 nutrition) or (naso-gastric* adj2 fed) or (naso-gastric* adj2 feed*) or (naso-gastric* adj2 therap*) or (naso-gastric* adj2 support*) or (naso-gastric* adj2 diet*) or (nasojejunal* adj2 nutrition) or (nasojejunal* adj2 fed) or (nasojejunal* adj2 feed*) or (nasojejunal* adj2 therap*) or (nasojejunal* adj2 support*) or (nasojejunal* adj2 diet*) or (naso-jejunal* adj2 nutrition) or (naso-jejunal* adj2 fed) or (naso-jejunal* adj2 feed*) or (naso-jejunal* adj2 therap*) or (naso-jejunal* adj2 support*) or (naso-jejunal* adj2 diet*)).tw,id. |
| 15 | ((ngt adj2 nutrition) or (ngt adj2 fed) or (ngt adj2 feed*) or (ngt adj2 therap*) or (ngt adj2 support*) or (ngt adj2 diet*)).tw,id. |
| 16 | ((njt adj2 nutrition) or (njt adj2 fed) or (njt adj2 feed*) or (njt adj2 therap*) or (njt adj2 support*) or (njt adj2 diet*)).tw,id. |
| 17 | ((non-oral adj2 nutrition) or (non-oral adj2 fed) or (non-oral adj2 feed*) or (non-oral adj2 therap*) or (non-oral adj2 support*) or (non-oral adj2 diet*)).tw,id. |
| 18 | ((nonoral adj2 nutrition) or (nonoral adj2 fed) or (nonoral adj2 feed*) or (nonoral adj2 therap*) or (nonoral adj2 support*) or (nonoral adj2 diet*)).tw,id. |
| 19 | ((orogastric* adj2 nutrition) or (orogastric* adj2 fed) or (orogastric* adj2 feed*) or (orogastric* adj2 therap*) or (orogastric* adj2 support*) or (orogastric* adj2 diet*) or (oro-gastric* adj2 nutrition) or (oro-gastric* adj2 fed) or (oro-gastric* adj2 feed*) or (oro-gastric* adj2 therap*) or (oro-gastric* adj2 support*) or (oro-gastric* adj2 diet*) or (orojejunal* adj2 nutrition) or (orojejunal* adj2 fed) or (orojejunal* adj2 feed*) or (orojejunal* adj2 therap*) or (orojejunal* adj2 support*) or (orojejunal* adj2 diet*) or (oro-jejunal* adj2 nutrition) or (oro-jejunal* adj2 fed) or (oro-jejunal* adj2 feed*) or (oro-jejunal* adj2 therap*) or (oro-jejunal* adj2 support*) or (oro-jejunal* adj2 diet*)).tw,id. |
| 20 | ((stomach adj2 nutrition) or (stomach adj2 fed) or (stomach adj2 feed*) or (stomach adj2 therap*) or (stomach adj2 support*) or (stomach adj2 diet*)).tw,id. |
| 21 | ((tube adj2 nutrition) or (tube adj2 fed) or (tube adj2 feed*) or (tube adj2 therap*) or (tube adj2 support*) or (tube adj2 diet*)).tw,id. |
| 22 | 7 or 8 or 9 or 10 or 11 or 12 or 13 or 14 or 15 or 16 or 17 or 18 or 19 or 20 or 21 |
| 23 | 6 and 22 |
| 24 | ((gastrostomy adj2 nutrition) or (gastrostomy adj2 fed) or (gastrostomy adj2 feed*) or (gastrostomy adj2 therap*) or (gastrostomy adj2 support*) or (gastrostomy adj2 diet*)).tw,id. |
| 25 | ((jejunostomy adj2 nutrition) or (jejunostomy adj2 fed) or (jejunostomy adj2 feed*) or (jejunostomy adj2 therap*) or (jejunostomy adj2 support*) or (jejunostomy adj2 diet*)).tw,id. |
| 26 | ((peg j adj2 nutrition) or (peg j adj2 fed) or (peg j adj2 feed*) or (peg j adj2 therap*) or (peg j adj2 support*) or (peg j adj2 diet*)).tw,id. |
| 27 | ((pegj adj2 nutrition) or (pegj adj2 fed) or (pegj adj2 feed*) or (pegj adj2 therap*) or (pegj adj2 support*) or (pegj adj2 diet*)).tw,id. |
| 28 | ((pej adj2 nutrition) or (pej adj2 fed) or (pej adj2 feed*) or (pej adj2 therap*) or (pej adj2 support*) or (pej adj2 diet*)).tw,id. |
| 29 | ((percutaneous endoscopic gastrostomy adj2 nutrition) or (percutaneous endoscopic gastrostomy adj2 fed) or (percutaneous endoscopic gastrostomy adj2 feed*) or (percutaneous endoscopic gastrostomy adj2 therap*) or (percutaneous endoscopic gastrostomy adj2 support*) or (percutaneous endoscopic gastrostomy adj2 diet*)).tw,id. |
| 30 | ((percutaneous endoscopic jejunostomy adj2 nutrition) or (percutaneous endoscopic jejunostomy adj2 fed) or (percutaneous endoscopic jejunostomy adj2 feed*) or (percutaneous endoscopic jejunostomy adj2 therap*) or (percutaneous endoscopic jejunostomy adj2 support*) or (percutaneous endoscopic jejunostomy adj2 diet*)).tw,id. |
| 31 | ((radiologically inserted gastrostomy adj2 nutrition) or (radiologically inserted gastrostomy adj2 fed) or (radiologically inserted gastrostomy adj2 feed*) or (radiologically inserted gastrostomy adj2 therap*) or (radiologically inserted gastrostomy adj2 support*) or (radiologically inserted gastrostomy adj2 diet*)).tw,id. |
| 32 | 24 or 25 or 26 or 27 or 28 or 29 or 30 or 31 |
| 33 | ((home or communit* or long term or longterm or residential* or outpatient or lifelong or life long or ambulatory) adj3 ((artificial* adj2 nutrition) or (artificial* adj2 fed) or (artificial* adj2 feed*) or (artificial* adj2 therap*) or (artificial* adj2 support*) or (artificial* adj2 diet*) or ((duoden* adj2 nutrition) or (duoden* adj2 fed) or (duoden* adj2 feed*) or (duoden* adj2 therap*) or (duoden* adj2 support*) or (duoden* adj2 diet*)) or ((enter* adj2 nutrition) or (enter* adj2 fed) or (enter* adj2 feed*) or (enter* adj2 therap*) or (enter* adj2 support*) or (enter* adj2 diet*)) or ((gastr* adj2 nutrition) or (gastr* adj2 fed) or (gastr* adj2 feed*) or (gastr* adj2 therap*) or (gastr* adj2 support*) or (gastr* adj2 diet*)) or ((intestinal* adj2 nutrition) or (intestinal* adj2 fed) or (intestinal* adj2 feed*) or (intestinal* adj2 therap*) or (intestinal* adj2 support*) or (intestinal* adj2 diet*)) or ((intragastr* adj2 nutrition) or (intragastr* adj2 fed) or (intragastr* adj2 feed*) or (intragastr* adj2 therap*) or (intragastr* adj2 support*) or (intragastr* adj2 diet*)) or ((jejun* adj2 nutrition) or (jejun* adj2 fed) or (jejun* adj2 feed*) or (jejun* adj2 therap*) or (jejun* adj2 support*) or (jejun* adj2 diet*)) or ((naso* adj2 nutrition) or (naso* adj2 fed) or (naso* adj2 feed*) or (naso* adj2 therap*) or (naso* adj2 support*) or (naso* adj2 diet*)) or ((ngt adj2 nutrition) or (ngt adj2 fed) or (ngt adj2 feed*) or (ngt adj2 therap*) or (ngt adj2 support*) or (ngt adj2 diet*)) or ((njt adj2 nutrition) or (njt adj2 fed) or (njt adj2 feed*) or (njt adj2 therap*) or (njt adj2 support*) or (njt adj2 diet*)) or ((non-oral adj2 nutrition) or (non-oral adj2 fed) or (non-oral adj2 feed*) or (non-oral adj2 therap*) or (non-oral adj2 support*) or (non-oral adj2 diet*)) or ((nonoral adj2 nutrition) or (nonoral adj2 fed) or (nonoral adj2 feed*) or (nonoral adj2 therap*) or (nonoral adj2 support*) or (nonoral adj2 diet*)) or ((oro* adj2 nutrition) or (oro* adj2 fed) or (oro* adj2 feed*) or (oro* adj2 therap*) or (oro* adj2 support*) or (oro* adj2 diet*)) or ((stomach adj2 nutrition) or (stomach adj2 fed) or (stomach adj2 feed*) or (stomach adj2 therap*) or (stomach adj2 support*) or (stomach adj2 diet*)) or ((tube adj2 nutrition) or (tube adj2 fed) or (tube adj2 feed*) or (tube adj2 therap*) or (tube adj2 support*) or (tube adj2 diet*)))).tw,id. |
| 34 | 23 or 32 or 33 |
| 35 | exp treatment planning/ or treatment guidelines/ or best practices/ or evidence based practice/ or professional standards/ or health care policy/ |
| 36 | (CPG* or guideline* or guidance or guide* or consensus or polic* or pathway* or recommendation* or regulation* or standard* or summar* or statement*).tw,id. |
| 37 | ((healthcare adj2 plan*) or (health care adj2 plan) or (treat* adj2 plan*)).tw,id. |
| 38 | (practice adj3 parameter*).tw,id. |
| 39 | ((position* adj2 paper) or (position* adj2 stand)).tw,id. |
| 40 | 35 or 36 or 37 or 38 or 39 |
| 41 | 34 and 40 |
| 42 | limit 41 to yr="2000 -Current |
| **CINAHL** | |
| S1 | (MH "Dietary Supplementation") |
| S2 | (MH "Enteral Nutrition") |
| S3 | (MH "Feeding Methods") |
| S4 | (MH "Feeding of Persons with Disabilities") |
| S5 | (MH "Feeding Tube Care+") |
| S6 | (MH "Feeding Tubes+") |
| S7 | (MH "Gastrostomy") |
| S8 | (MH "Home Nutritional Support") |
| S9 | (MH "Jejunostomy") |
| S10 | (MH "Nutritional Support") |
| S11 | ti (("artificial*" OR "duoden*" OR "enter*" OR "gastr*" OR "intestinal*" OR "intragastr*" OR "jejun*" OR "naso-gastric*" OR "nasogastric*" OR "naso-jejun*" OR "nasojejun*"OR "oro-gastric*" OR "orogastric*" OR "oro-jejun*" OR "orojejun*" OR "ngt" OR "njt" OR "non oral" OR "nonoral" OR "oro*" OR OR "stomach" OR "tube") N2 ("nutrition" OR "fed" OR "feed*" OR "therap*" OR "support*" OR "diet*")) |
| S12 | AB (("artificial*" OR "duoden*" OR "enter*" OR "gastr*" OR "intestinal*" OR "intragastr*" OR "jejun*" OR "naso-gastric*" OR "nasogastric*" OR "naso-jejun*" OR "nasojejun*" OR "oro-gastric*" OR "orogastric*" OR "oro-jejun*" OR "orojejun*" OR "ngt" OR "njt" OR "non oral" OR "nonoral" OR "oro*" OR OR "stomach" OR "tube") N2 ("nutrition" OR "fed" OR "feed*" OR "therap*" OR "support*" OR "diet*")) |
| S13 | S1 OR S2 OR S3 OR S4 OR S5 OR S6 OR S7 OR S8 OR S9 OR S10 OR S11 OR S12 |
| S14 | ti ((“gastrostomy” OR "jejunostomy" OR "peg j" OR "pegj" OR "pej" OR “percutaneous endoscopic gastrostomy” OR “percutaneous endoscopic jejunostomy” OR “radiologically inserted gastrostomy”) N2 ("nutrition" OR "fed" OR "feed*" OR "therap*" OR "support*" OR "diet*")) |
| S15 | AB ((“gastrostomy” OR "jejunostomy" OR "peg j" OR "pegj" OR "pej" OR “percutaneous endoscopic gastrostomy” OR “percutaneous endoscopic jejunostomy” OR “radiologically inserted gastrostomy”) N2 ("nutrition" OR "fed" OR "feed*" OR "therap*" OR "support*" OR "diet*")) |
| S16 | S14 OR S15 |
| S17 | (MH "Community Health Centers") |
| S18 | (MH "Community Health Nursing") |
| S19 | (MH "Community Health Services") |
| S20 | (MH "Community Living+") |
| S21 | (MH "Home Health Aides") |
| S22 | (MH "Home Health Care") |
| S23 | (MH "Home Nursing, Professional") |
| S24 | (MH "Home Nursing") |
| S25 | (MH "Home Nutritional Support") |
| S26 | (MH "Home Rehabilitation") |
| S27 | (MH "Home Visits") |
| S28 | (MH "Long Term Care") |
| S29 | (MH "Nursing Home Patients") |
| S30 | (MH "Rehabilitation, Community-based") |
| S31 | (MH "Respite Care") |
| S32 | S17 OR S18 OR S19 OR S20 OR S21 OR S22 OR S23 OR S24 OR S25 OR S26 OR S27 OR S28 OR S29 OR S30 OR S31 |
| S33 | S13 AND S32 |
| S34 | TI (("home" OR "community" OR "long term" OR "longterm" OR "residential" OR "outpatient" OR “lifelong” OR "life long" OR "ambulatory") N3 (("artificial*" OR "duoden*" OR "enter*" OR "gastr*" OR "intestinal*" OR "intragastr*" OR "jejun*" OR "naso-gastric*" OR "nasogastric*" OR "naso-jejun*" OR "nasojejun*" OR "oro-gastric*" OR "orogastric*" OR "oro-jejun*" OR "orojejun*" OR "ngt" OR "njt" OR "non oral" OR "nonoral" OR "oro*" OR "stomach" OR "tube") N2 ("nutrition" OR "fed" OR "feed*" OR "therap*" OR "support*" OR "diet*"))) |
| S35 | AB (("home" OR "community" OR "long term" OR "longterm" OR "residential" OR "outpatient" OR “lifelong” OR "life long" OR "ambulatory") N3 (("artificial*" OR "duoden*" OR "enter*" OR "gastr*" OR "intestinal*" OR "intragastr*" OR "jejun*" OR "naso-gastric*" OR "nasogastric*" OR "naso-jejun*" OR "nasojejun*" OR "oro-gastric*" OR "orogastric*" OR "oro-jejun*" OR "orojejun*" OR "ngt" OR "njt" OR "non oral" OR "nonoral" OR "oro*" OR "stomach" OR "tube") N2 ("nutrition" OR "fed" OR "feed*" OR "therap*" OR "support*" OR "diet*"))) |
| S36 | S34 OR S35 |
| S37 | S16 OR S33 OR S36 |
| S38 | (MH "Adult+") |
| S39 | ti (adult* or m#n or wom#n or elder* or senior* or (old* adj (person* or people*))) |
| S40 | AB (adult* or m#n or wom#n or elder* or senior* or (old* adj (person* or people*))) |
| S41 | S38 OR S39 OR S40 |
| S42 | S37 AND S41 |
| S43 | (MH "Health Policy") |
| S44 | (MH "Practice Guidelines") |
| S45 | (MH "Nursing Protocols") |
| S46 | (MH "Protocols") |
| S47 | (MH "Hospital Policies") |
| S48 | AB ("CPG*" or "guideline*" or "guidance" or "guide*" or "consensus" or "polic*" or "pathway*" or "recommendation*" or "regulation*" or "standard*" or "summar*" or "statement*") |
| S49 | TI ("CPG*" or "guideline*" or "guidance" or "guide*" or "consensus" or "polic*" or "pathway*" or "recommendation*" or "regulation*" or "standard*" or "summar*" or "statement*") |
| S50 | TI ((practice N3 parameter*) or (position* N2 (paper adj2 stand))) |
| S51 | (MH "Professional Practice, Evidence-Based+") |
| S52 | (MH "Delphi Technique") |
| S53 | TI((healthcare or health care or treat*) N2 plan*) |
| S54 | AB((healthcare or health care or treat*) N2 plan*) |
| S55 | AB ((practice N3 parameter*) or (position* N2 (paper adj2 stand))) |
| S56 | S43 OR S44 OR S45 OR S46 OR S47 OR S48 OR S49 OR S50 OR S51 OR S52 OR S53 OR S54 OR S55 |
| S57 | S42 AND S56 – limit publication to after 20000101 |
| **Scopus** | |
| 1 | ( TITLE-ABS-KEY ( ( "home" OR "community" OR "longterm" OR "long term" OR "residential*" OR "outpatient" OR "lifelong" OR "life long" OR "rehabilitation" OR "ambulatory" ) W/3 ( ( "artificial*" OR "duoden*" OR "enteric" OR "enteral*" OR "gastr*" OR "intestinal*" OR "intragastr*" OR "jejun*" OR "nasogastric*" OR "naso-gastric*" OR "nasojejunal*" OR "naso-jejunal*" OR "orogastric*" OR "oro-gastric*" OR "orojejunal*" OR "oro-jejunal*" OR "ngt" OR "njt" OR "non oral" OR "non-oral" OR "nonoral" OR "stomach" OR "tube" ) W/2 ( "nutrition" OR "fed" OR "feed*" OR "therap*" OR "support*" OR "diet*" ) ) ) OR TITLE-ABS-KEY ( ( "gastrostomy" OR "jejunostomy" OR "peg j" OR "peg-j" OR "pegj" OR "pej" OR "percutaneous endoscopic gastrostomy" OR "percutaneous endoscopic jejunostomy" OR "radiologically inserted gastrostomy" ) W/3 ( "nutrition" OR "fed" OR "feed*" OR "therap*" OR "support*" OR "diet*" ) ) ) AND ( TITLE-ABS-KEY ( "adult*" OR "m?n" OR "wom?n" OR "elder*" OR "senior*" OR "middle aged" OR "geriatric*" OR ( "old*" W/1 ( "person*" OR "people*" OR "population*" OR "subject*" OR "female*" OR "male*" OR "patient*" ) ) ) ) AND ( TITLE-ABS-KEY ( "CPG*" OR "guideline*" OR "guidance" OR "guide*" OR "consensus" OR "polic*" OR "pathway*" OR "recommendation*" OR "regulation*" OR "standard*" OR "summar*" OR "statement*" OR "delphi" OR "practice parameter*" ) ) AND PUBYEAR > 1999 AND ( LIMIT-TO ( LANGUAGE , "English" ) ) |
