# Supplementary material for: A systematic review and quality appraisal of guidelines and recommendations for home enteral tube feeding in adults
Source: Eur J Clin Nutr. 2024 Sep 3;79(2):104–12. doi: 10.1038/s41430-024-01500-1 (PMC11810790; doi:10.1038/s41430-024-01500-1)
Supplement: Supplementary file 2 — Supplementary Material_Table S2-S6 [file 41430_2024_1500_MOESM2_ESM.pdf]

Table S2. Grey Literature Sources Searched and Methods Used.

Source Type	Guideline Repositories
Methods	Repositories were searched using the search functions available and keywords from the bibliographical search strategy (Table S1)
Repositories Included	• DynaMed*<br>• ECRI Guidelines Trust<br>• Guidelines International Network (GIN) - Powered by EBSCO<br>• Index Medicus (multiple Regions)<br>• National Guideline Clearinghouse (NGC) via The Alliance for the Implementation of Clinical Practice Guidelines<br>• National Institute for Health and Care Excellence (NICE) Guidance<br>• Trip
Source Type	Websites
Methods	1) When a search function was available, websites were searched using keywords from the bibliographical search strategy (Table S1).<br>a. Where there were >100 results for an in-website search, the results were sorted by relevance before screening.<br>b. Screening stopped when irrelevant records (not enteral feeding-related) consistently appeared or when the first 100 records were screened, whichever occurred first.<br>2) If no search function was available, a manual search of the website was conducted navigating through each webpage.
Websites Included	• Agency for Clinical Innovagtion (ACI) - https://aci.health.nsw.gov.au/<br>• ASPEN† - https://www.nutritioncare.org/<br>• AusPEN† - https://www.auspen.org.au/<br>• BAPEN† - https://www.bapen.org.uk/<br>• Commission on Excellence and Innovation in Health (CEIH) - https://ceih.sa.gov.au/<br>• ESPEN† - https://www.espen.org/<br>• Government of South Australia (SA Health) - https://www.sahealth.sa.gov.au/<br>• Health Service Executive (HSE) Ireland - https://www.hse.ie/<br>• IrSPEN - https://irspen.ie/<br>• National Dietetics Associations listed as Members of the International Confederation of Dietetic Associations (ICDA) † (49 Websites) - https://internationaldietetics.org/<br>• National Institute for Health and Care Research (NIHCR) - https://www.nihr.ac.uk/<br>• PENG - https://www.peng.org.uk/<br>• Practice-based Evidence in Nutrition (PEN) - https://www.pennutrition.com/<br>• Queensland Health - https://www.health.qld.gov.au/<br>• United Healthcare Community Plan - https://www.uhc.com/communityplan<br>• Western Australia (WA) Country Health Service - https://www.wacountry.health.wa.gov.au/

* Authors had incomplete access

† Authors had membership access to these websites and ICDA member Dietitians Australia only.

Table S3. Clusters used in AGREE-REX quality appraisal.

Cluster	Details
Commencement of HETF	Recommendations included were relevant to assessment & diagnosis of HETF candidates including choosing the enteral route and indications and contraindications of HETF
Care of Feeding Site and Enteral Access Devices	Recommendations included were relevant to choice and care of enteral access devices and stoma sites e.g. to reduce site complications
Recommendations for Feeding	Recommendations were related to feeding regimes and nutritional requirements
Monitoring HETF	Recommendations were related to outcomes to be collected over the patient journey, HETF reassessment and review timeframes
Termination of HETF	Recommendations related to cessation of HETF
Requirements for HETF Services	Recommendations included relate to HETF service requirements e.g. staffing, roles, education

Table S4. AGREE-II consensus item scores for included guidelines (n=15).

**Domain**	**1**			**2**			**3**								**4**			**5**				**6**	
**Item**	**1**	**2**	**3**	**4**	**5**	**6**	**7**	**8**	**9**	**10**	**11**	**12**	**13**	**14**	**15**	**16***	**17**	**18**	**19**	**20**	**21**	**22†**	**23**
NICE (12)	7	7	7	7	7	7	6	6	7	6	7	7	6	7	6	7	7	1	5	6	6	6	4
ESPEN (35)	7	7	7	5	1	6	5	7	7	7	7	7	2	6	6	7	7	2	2	1	2	2	4
ESPEN (4)	6	7	5	5	3	7	5	3	7	7	7	7	2	6	5	6	7	1	2	1	2	3	4
NSW ACI (40)	6	7	7	6	3	7	4	4	5	4	6	6	5	4	5	7	6	1	4	1	2	1	1
KSGE (36)	5	7	5	3	1	6	3	4	6	7	6	7	1	3	6	5	7	2	1	1	1	6	3
ESGE (37,38)	5	5	6	4	1	2	3	4	4	4	7	5	1	4	6	7	6	1	1	1	1	2	3
AIOSS, ANOTE, ANIGEA (39)	7	6	6	4	2	5	4	4	3	2	3	4	3	1	5	6	3	1	1	1	1	4	2
ASPEN (32)	6	5	7	5	1	6	1	1	5	2	6	7	7	2	6	7	6	1	2	1	1	1	1
ASPEN (31)	6	6	4	4	1	6	1	2	2	3	7	5	2	1	5	7	6	1	1	1	1	2	3
HAS (33)	6	7	7	4	1	6	3	4	2	3	3	1	4	1	4	7	2	1	3	1	1	1	2
ECFS (30)	6	5	5	5	3	7	3	1	1	6	3	3	2	1	4	6	5	1	2	1	2	3	3
NSW ACI (29)	6	5	7	4	1	7	3	2	2	2	3	2	3	1	4	5	3	1	3	1	3	1	1
BSG (41)	6	4	5	4	1	7	1	1	2	1	5	4	1	1	5	7	6	2	4	1	1	1	2
CREST (28)	4	4	3	5	2	3	1	1	1	1	2	2	1	1	4	5	4	3	2	1	1	1	1
ASPEN (34)	5	4	6	3	1	5	1	1	1	1	2	2	1	1	4	6	6	1	1	1	1	2	1
Median (IQR)	6 (5.5-6)	6 (5-7)	6 (5-7)	4 (4-5)	1 (1-2.5)	6 (5.5-7)	3 (1-4)	3 (1-4)	3 (2-5.5)	3 (2-6)	6 (3-7)	5 (2.5-7)	2 (1-3.5)	1 (1-4)	5 (4-6)	7 (6-7)	6 (4.5-6.5)	1 (1-1.5)	2 (1-3)	1 (1-1)	1 (1-2)	2 (1-3)	2 (1-3)

*In scoring item 16, alternative treatments were considered different nutrition support options and different feeding devices dependent on the scope of the guideline.

† Influence of the funding body on the guidelines was not as applicable for many of these guidelines as they were developed and funded by professional associations interested in generating guidelines to improve patient management, thus in certain instances this item was graded 1 (absence of information).

Abbreviations: AIOSS - Italian Association of Stoma care Nurses; ANIGEA - Italian Association of Gastroenterology Nurses and Associates; ANOTE - Italian Association of Endoscopic Operators; ASPEN - American Society for Clinical Nutrition and Metabolism; BSG – British Society of Gastroenterology; CREST - Clinical Resource Efficiency Support Team; ECFS - European Cystic Fibrosis Society; ESGE - European Society of Gastrointestinal Endoscopy; ESPEN - European Society for Clinical Nutrition and Metabolism, GENCA - Gastroenterological Nurses College of Australia; HAS- Haute Autorité de santé (French National Authority for Health); KSGE - Korean Society of Gastrointestinal Endoscopy; NICE - National Institute for Health and Care Excellence; NSW ACI – New South Wales Agency for Clinical Innovation.

Table S5. Summary of total and cluster recommendation counts further categorised by their assigned grading.

	Guideline			All Guidelines
	NICE (12)	ESPEN (35)	ESPEN (4)	
All Recommendations	29	19	61	109
A	2 (7%)	1 (5%)	3 (5%)	6 (6%)
B	1 (3%)	4 (21%)	15 (25%)	20 (18%)
O	n/a		11 (18%)	11 (10%)
GPP	26 (90%)	14 (74%)	32 (52%)	72 (66%)
Recommendation Cluster				
Commencement of HETF	4	5	7	16
A	1			1 (6%)
B			2	2 (13%)
GPP	3	5	5	13 (81%)
Care of Feeding Site and Enteral Access Devices	6	2	30	38
A	1		2	3 (8%)
B			5	5 (13%)
O			9	9 (24%)
GPP	5	2	14	21 (55%)
Recommendations for Feeding	3	7	10	20
A			1	1 (5%)
B	1	3	2	6 (30%)
O			2	2 (10%)
GPP	2	4	5	11 (55%)
Monitoring HETF	3	2	5	10
B			1	1 (10%)
GPP	3	2	4	9 (90%)
Termination of HETF	1	N/A	1	2
GPP	1		1	2 (100%)
Requirements for HETF Services	12	3	8	23
A		1		1 (4%)
B		1	5	6 (26%)
GPP	12	1	3	16 (70%)

All three guidelines used the Scottish Intercollegiate Guidelines Network level of evidence classification system and a comparable recommendation grading system therefore original grading by the guideline development groups was preserved. Grade A: At least one meta-analysis, systematic review, or RCT rated as 1++, and directly applicable to the target population; or a body of evidence consisting principally of studies rated as 1+, directly applicable to the target population, and demonstrating overall consistency of results. NICE also included 'Evidence drawn from a NICE technology appraisal' to this category although this was not relevant to the recommendations included in this assessment. Grade B: A body of evidence including studies rated as 2++, directly applicable to the target population; or A body of evidence including studies rated as 2+, directly applicable to the target population and demonstrating overall consistency of results; or Extrapolated evidence from studies rated as 1++ or 1+. The NICE guideline only included evidence rated as 1- or higher and so grade B recommendations from this document were based on extrapolated evidence from higher level evidence. Grade O: Evidence level 3 or 4; or Extrapolated evidence from studies rated as 2++ or 2+. GPP (Good Practice Point): Recommended best practice based on the clinical experience of the guideline development group. Abbreviations: ESPEN - European Society for Clinical Nutrition and Metabolism; NICE - National Institute for Health and Care Excellence; IQR - Interquartile Range.

Table S6. AGREE-REX Consensus Item Scores for all six recommendation clusters for all included guidelines (n=3).

Guideline	Recommendation Cluster†	AGREE-REX Consensus Item* Scores (/7)									Recommendation
		1	2	3	4	5	6	7	8	9	
NICE (12)	A	5	7	2	3	3	2	2	4	3	Yes, with modifications
	B	4	7	3	3	3	4	2	4	3	Yes, with modifications
	C	4	7	4	4	4	1	2	4	2	Yes, with modifications
	D	1	5	2	2	2	1	2	4	3	Yes, with modifications
	E	1	4	1	2	2	1	2	4	2	Yes, with modifications
	F	3	6	4	2	5	3	2	5	4	Yes, with modifications
ESPEN (4)	A	3	5	4	3	4	1	1	4	1	Yes, with modifications
	B	4	6	4	3	4	2	1	4	1	Yes, with modifications
	C	4	6	3	3	4	1	1	4	1	Yes, with modifications
	D	3	6	5	3	3	1	1	4	1	Yes, with modifications
	E	1	5	3	3	2	1	1	4	1	Yes, with modifications
	F	3	4	3	1	1	3	1	4	2	Yes, with modifications
ESPEN (35)	A	1	6	3	3	3	1	1	4	2	Yes, with modifications
	B	1	6	2	3	3	1	2	4	1	Yes, with modifications
	C	4	7	3	3	2	1	2	4	1	Yes, with modifications
	D	1	6	3	3	4	2	1	4	2	Yes, with modifications
	F	5	5	4	3	2	3	1	4	2	Yes, with modifications
Median Item Score (IQR)		3 (1-4)	6 (5-6)	3 (3-4)	3 (3-3)	3 (2-4)	1 (1-2)	1 (1-2)	4 (4-4)	2 (1-2)	

*Items: 1 – Evidence; 2 – Applicability to Target Users; 3 – Applicability to Patients/Populations; 4 – Values and Preferences of Target Users; 5 - Values and Preferences of Patients/Populations; 6 - Values and Preferences of Policy/Decision-Makers; 7 - Values and Preferences of Guideline Developers; 8 – Purpose; 9 – Local Application and Adoption

† Recommendation Clusters: A- Commencement of HETF; B - Care of Feeding Site and Enteral Access Devices; C - Recommendations for HETF Feeds; D - Monitoring HETF E - Termination of HETF; F - Requirements for HETF Services.

Abbreviations: ESPEN - European Society for Clinical Nutrition and Metabolism; NICE - National Institute for Health and Care Excellence; IQR - Interquartile Range.
